# Plasma functionalization for cyclic transition between neutral and charged excitons in monolayer MoS_2_

**DOI:** 10.1038/srep21405

**Published:** 2016-02-22

**Authors:** Y. Kim, Y. I. Jhon, J. Park, C. Kim, S. Lee, Y. M. Jhon

**Affiliations:** 1 Sensor System Research Center, Korea Institute of Science and Technology, Seoul 136-791, Republic of Korea

## Abstract

Monolayer MoS_2_ (1L-MoS_2_) has photoluminescence (PL) properties that can greatly vary via transition between neutral and charged exciton PLs depending on carrier density. Here, for the first time, we present a chemical doping method for reversible transition between neutral and charged excitons of 1L-MoS_2_ using chlorine-hydrogen-based plasma functionalization. The PL of 1L-MoS_2_ is drastically increased by p-type chlorine plasma doping in which its intensity is easily tuned by controlling the plasma treatment duration. We find that despite their strong adhesion, a post hydrogen plasma treatment can very effectively dedope chlorine adatoms in a controllable way while maintaining robust structural integrity, which enables well-defined reversible PL control of 1L-MoS_2_. After exhaustive chlorine dedoping, the hydrogen plasma process induces n-type doping of 1L-MoS_2_, degrading the PL further, which can also be recovered by subsequent chlorine plasma treatment, extending the range of tunable PL into a bidirectional regime. This cyclically-tunable carrier doping method can be usefully employed in fabricating highly-tunable n- and p-type domains in monolayer transition-metal dichalcogenides suitable for two-dimensional electro-optic modulators, on-chip lasers, and spin- and valley-polarized light-emitting diodes.

Recently, two-dimensional (2D) transition-metal dichalcogenides (TMDs)^1^,^2^, which consist of hexagonally arranged transition metal atoms (Mo, W, Sn) that are sandwiched between two layers of chalcogen atoms (S, Se, Te), have gained great attention for electronic and photonic applications due to their superb optoelectronic properties. Many of them have sizable electronic band gaps that undergo indirect-to-direct transition when the thickness is reduced to a monolayer^3^,^4^,^5^,^6^. They have inversion symmetry breaking together with spin-orbit coupling providing a novel platform for the study of valleytronics^7^,^8^. They also have saturable absorber properties that are suitable for passively Q-switched laser applications for femtosecond laser generation, pulse modulators, and optical limiters in wave guiding photonic devices^9^,^10^,^11^,^12^,^13^. In particular, the reduced dielectric screening in the 2D materials and the heavy effective mass of charge carriers arising from *d*-orbitals of the transition-metal atoms significantly increase the Coulombic interaction between the electrons and holes that are optically generated in the monolayer TMDs (1L-TMDs), creating stable electron-hole pairs (neutral excitons) even at room temperature^14^,^15^. These excitons can further become charged by binding an additional electron or hole to form charged excitons named trions.

1L-TMDs exhibit a significantly lower photoluminescence (PL) than that expected in high-quality direct band-gap semiconductors due to the abundance of trions in naturally n-doped TMDs^16^,^17^. This low PL intensity of 1L-TMDs can be substantially enhanced by decreasing the electron density and thus inducing the trion-to-exciton transition^16^,^18^,^19^. So far, gate voltage application^16^ and gas physorption^18^ have been suggested as charge doping methods to control the PL intensity of TMDs. However, the requirements of the complicated structure of field effect transistors (FETs) and the precise control of gas regulation have limited the applicability of these methods. In addition, the effect of gate electric field on the transport properties of MoS_2_-FETs significantly varies depending on the gate bias polarity due to the charge trapping via the adsorption or desorption of oxygen and water on the MoS_2_ surface under a positive or negative gate bias, respectively, at an ambient environment^20^.

Recently, Mauri *et al.* suggested the use of solution-based chemical doping as an efficient and convenient means to control the PL properties of 1L-MoS_2_^19^. They showed that the PL intensity of 1L-MoS_2_ can increase or decrease depending on whether p-type dopants of 2,3,5,6-tetrafluoro-7,7,8,8-tetracyanoquinodimethane (F_4_TCNQ), or n-type dopants of nicotinamide adenine dinucleotide (NADH) cover the surface. However, despite its compactness, the volatile nature of F_4_TCNQ molecules and a large quantity of surface coverage required for proper PL modulation have raised serious concerns with respect to thermal stability, cross-contamination, molecular sensing capability, and reversible PL control^21^.

In this article, we have shown that chlorine-hydrogen-based plasma functionalization can offer the most efficient and reliable means to reversibly tune the PL properties of 1L-MoS_2_ in the chemical doping regime. The PL of 1L-MoS_2_ is drastically increased through chlorine plasma doping, and its intensity can be largely tuned by adjusting the plasma treatment time. We find that this enhanced PL can be easily reduced back to the original luminescence level of the as-prepared 1L-MoS_2_ by applying a post hydrogen plasma treatment. The X-ray photoelectron spectroscopy (XPS) measurements indicate that it is a consequence of efficient dedoping of chlorine adatoms in which the dedoping level can be controlled by regulating the hydrogen plasma treatment time. After complete elimination of chlorine atoms, this hydrogen plasma treatment leads to n-type doping of 1L-MoS_2_, decreasing the PL intensity further below the luminescence level of as-prepared 1L-MoS_2_. This degradation in the PL intensity can also be recovered through subsequent chlorine plasma treatment. Density functional theory (DFT) calculations show that despite the strong adhesion of the chlorine (or hydrogen) atoms, hydrogen (or chlorine)-plasma-assisted chlorine (or hydrogen)-dedoping reaction readily occurs with a negligible energy barrier and a large negative reaction energy that is 2 to 3 times as large as the adsorption energy of the chlorine (or hydrogen) atoms.

## Results

### Enhancement of PL in MoS_2_ via chlorine plasma doping

**Figure 1:**
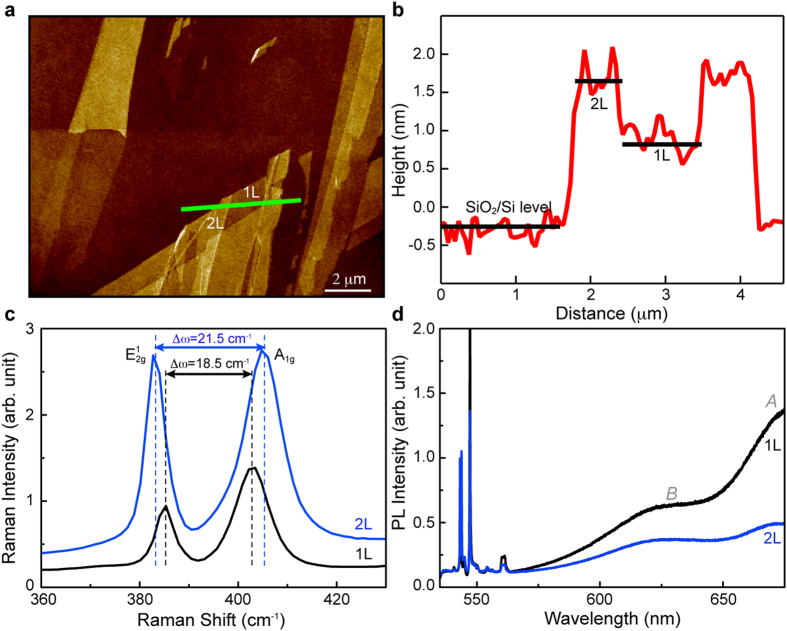
AFM image and Raman and PL spectra of 1L- and 2L-MoS_2_. (**a**) AFM image of the MoS_2_ flake. (**b**) Height profile of the MoS_2_ taken along the green solid line of **a**. (**c**) Raman and (**d**) PL spectra measured for 1L- and 2L-MoS_2_.

The number of layers of MoS_2_ is determined by atomic force microscopy (AFM) (Fig. 1a). The variation in AFM height profile that is measured along the green solid line in Fig. 1a indicates that the step height of MoS_2_ is ~0.7 nm (Fig. 1b), which is comparable to the monolayer thickness of ~0.6 nm in the MoS_2_ crystal^22^ and their small difference results from a non-contact mode of the AFM scanning. The Raman and PL spectroscopies confirm the presence of the mono- and bi-layer (2 L) MoS_2_ domains that are identified above. The frequency difference between the 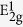
(in-plane) and 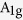
(out-of-plane) Raman modes is measured as ~18.5 and ~21.5 cm^−1^ for the 1 L- and 2L-MoS_2_, respectively, showing excellent agreement with the results of previous studies^23^,^24^. The PL spectrum of MoS_2_ has two distinct PL bands at ~680 and ~625 nm that are respectively denoted as *A* and *B* in Fig. 1d. These PL bands are associated with the direct transition at the *K* and *K’* points in the Brillouin zone, respectively, and their energy difference corresponds to valence-band splitting resulting from the strong spin-orbit interaction^3^,^4^. The intensity of *A* peak remarkably increases in the 1 L-MoS_2_ compared to that of 2 L-MoS_2_ due to the indirect-to-direct band-gap transition (Fig. 1d). These PL results are also in good agreement with previous results^3^,^4^.

**Figure 2:**
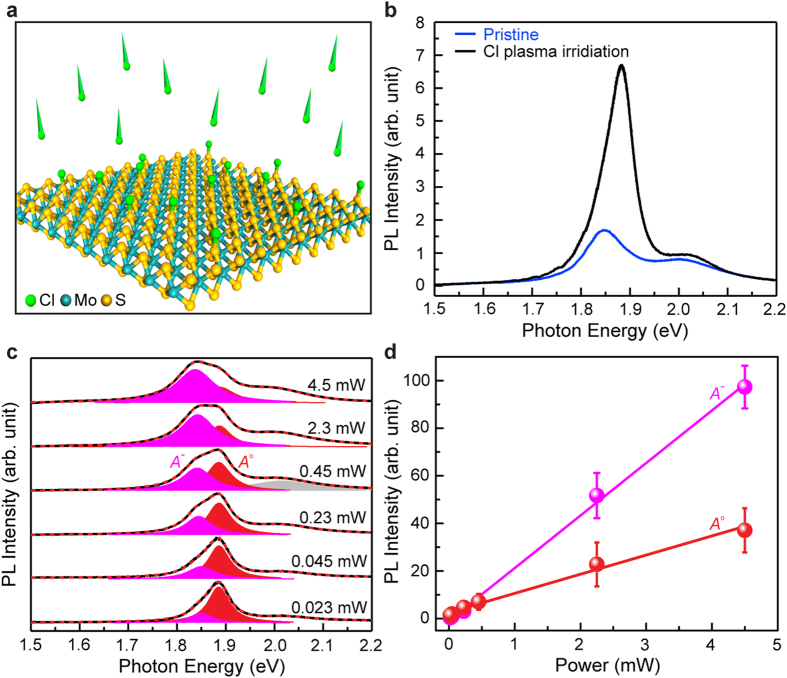
PL spectrum of chlorine-doped 1L-MoS_2_ with laser power variation. (**a**) Schematic representation of chlorine plasma doping onto the surface of 1L-MoS_2_. (**b**) The PL spectra of the chlorine-doped (black) and undoped (blue) 1L-MoS_2_ in which the plasma treatment time of 14 sec and the RF power of 5 W were used for chlorine plasma doping. (**c**) The PL spectral evolution of 1L-MoS_2_ (normalized by the maximum intensity in each case) as the laser power increases. The typical multiple Lorentzian fitting is illustrated in the case of the 0.45 mW laser power. (**d**) The PL intensities of the excitons (*A*^o^) and trions (*A*^−^) as a function of the laser power.

This verified monolayer sample is used to investigate how chlorine plasma doping affects the PL properties of 1L-MoS_2_. The typical plasma doping process employed in this study is illustrated in Fig. 2a. To make it clear that chlorine atoms are doped on 1 L-MoS_2_ after chlorine plasma treatment, we performed AFM analysis on plasma treated 1 L-MoS_2_ and pristine 1 L-MoS_2_, in which distinct increases in lateral thickness were observed in plasma treated 1 L-MoS_2_ compared to pristine one (Supplementary Fig. 1)

We find that the weak PL intensity of the *A* band in undoped 1L-MoS_2_ is greatly enhanced by chlorine plasma doping (Fig. 2b) due to the change from trion PL to exciton PL^16^,^17^. We also find that the chlorine-doped MoS_2_ exhibits a good ambient stability as seen in its consistent PL behaviors even after one month in the ambient environment (Supplementary Fig. 2). The *A* band is composed of *A*^−^ and *A*^o^ peaks that respectively correspond to the exciton (~1.84 eV) and trion resonances (~1.88 eV) (Fig. 2c).

Multiple Lorentzian fits are used to extract their respective spectral weights from the PL spectrum of 1L-MoS_2_. The PL spectrum of MoS_2_ is well reproduced by the sum (red dashed line) of the *A*^−^ and *A*^o^ peak components (red and magenta areas) and the *B* exciton peak component (gray colored area), as representatively shown in the case where a laser power of 0.45 mW is used for the excitation in Fig. 2c.

In general, the PL intensity is described by *I* ~ *L*^*k*^ where *I* is the PL intensity and *L* is the laser excitation power, and the power value *k* is close to 1 for the exciton and trion PLs as a consequence of their direct recombination^25^. We find that the integrated PL intensities of trions (magenta) and excitons (red) in chlorine-doped 1L-MoS_2_ are in a good linear correlation with the laser power, as expected, and the slope of the trions is larger than that of the excitons (Fig. 2d). Such linear power dependence of exciton and trion PL has also been observed in other 2D materials^26^,^27^.

**Figure 3:**
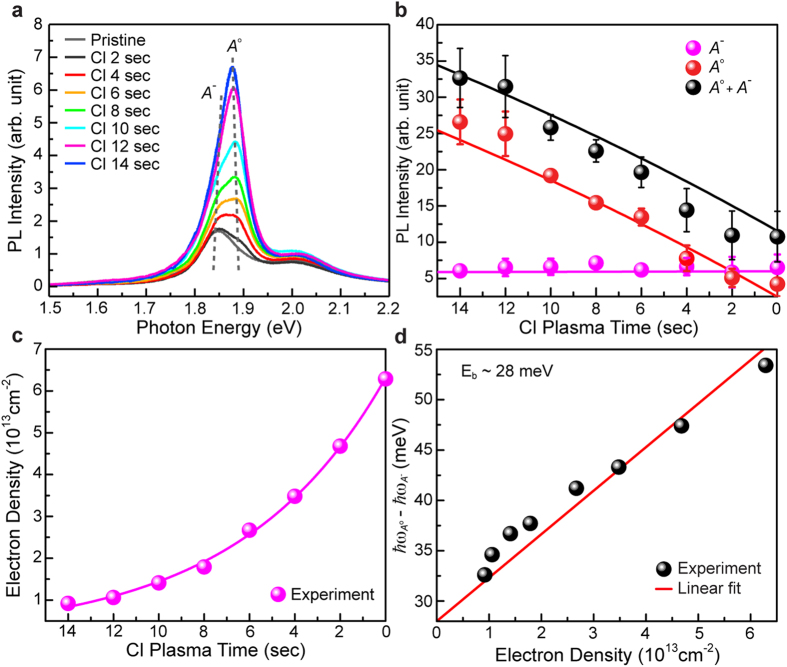
The variation of PL properties of 1L-MoS_2_ according to the duration of the chlorine plasma treatment. (**a**) Evolution of the PL spectrum of 1L-MoS_2_ as the chlorine plasma treatment time increases under the RF power of 5 W. The resonance energies of the excitons (*A*^o^) and the trions (*A*^−^) are indicated with gray dotted lines. (**b**) The PL intensities of the excitons, trions, and their total contribution as a function of the plasma treatment time. (**c**) Electron density as a function of the plasma treatment time. (**d**) The difference between the exciton and trion energies as a function of the electron density. These parameters conform to a good linear correlation, and its linear fit (red line) has an intercept of ~28 meV which determines the trion binding energy of 1L-MoS_2_.

Next, we have investigated how the PL properties of 1L-MoS_2_ change as the plasma treatment time (i.e., the chlorine doping level) varies while all the other conditions remain fixed during the process (see the method section). Figure 3a shows that the PL intensity of 1L-MoS_2_ monotonically increases as the plasma treatment time increases up to 14 sec, and the enhanced PL intensity is maximally ~ 3 times that of undoped 1L-MoS_2._ The spectral shape of the PL in MoS_2_ also undergoes a significant variation as the plasma treatment time increases. Specifically, the peak of the total PL spectrum gradually blueshifts with increasing the plasma treatment time due to a dominant increase in the intensity of the *A*^o^ peak (Fig. 3a). The chlorine plasma functionalization can also affect the Raman spectrum since the zone center phonons in MoS_2_ likely change depending on the amount of adsorbates^28^,^29^. Actually, we find that the 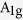 phonon stiffens in 1L-MoS_2_ as the plasma treatment time increases while the 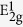 phonon remains almost intact (Supplementary Fig. 3). However, after the plasma time of 14 sec, the PL signal is gradually quenched (Supplementary Fig. 4a), which is inferred to result from the occurrence of conceivable plasma etching^30^. The Raman signals of the sample are significantly suppressed after this threshold time (Supplementary Fig. 4b), supporting the above conjecture.

We then perform an in-depth analysis on the variation of the exciton and trion PL intensities as the plasma treatment time increases by decomposing the PL spectra into the corresponding spectral components. Fig. 3b shows that the PL intensity of the trions is larger than that of the excitons in the undoped 1L-MoS_2_ since as-prepared 1L-MoS_2_ is generally heavily electron-doped^16^,^19^. However, as the 1L-MoS_2_ is counter-doped by chlorine, the PL intensity of the excitons (*I*_*A°*_: red circles) increases up to 4 to 5 times the initial value while the PL intensity of trions (*I*_*A*^−^_: magenta circles) remains almost constant. As a consequence, the total PL intensity (*I*_total_: black circles) of 1L-MoS_2_ is dominated by the exciton PL when sufficient charge neutralization has been achieved.

Assuming that the mass action law is valid^17^, we obtain a relationship among the trion PL spectral weight (*I*_A^−^_/*I*_total_), the electron carrier density (*n*_el_, unit: electron number/cm^2^), and the trion binding energy (*E*_b_) in 1L-MoS_2_ (see the method section) as shown below:


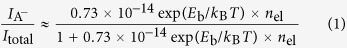


where *k*_B_ is the Boltzmann constant, *T* is the temperature, set to be 300 K here, and *E*_b_ is the trion binding energy that is taken to be 20 meV as an initial guessed value temporarily.

In the regime of a 2D electron gas model (*E*_F_ = *ħ*^*2*^*πn*_el_/*m*_e_), the splitting between the exciton and trion energies in 1L-MoS_2_ is linearly dependent on the electron density as^31^:


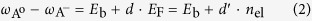


where 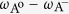 is the splitting between the exciton and trion energies, 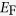 is the Fermi energy, and *d* is a fitting parameter. Our result is described by equation (2) very well, and its linear fit (red line) has an intercept of ~28 meV (Fig. 3d). At charge-neutral condition (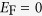), the splitting between the exciton and trion energies is the same as the trion binding energy *E*_b_, which is the minimum energy that is required to promote one electron in a trion to the conduction band. Therefore, the intercept of the linear fit determines the trion binding energy of 1L-MoS_2_. If this trion binding energy differs from the value (20 meV) that was initially guessed, we use it as a new input for the trion binding energy in equation (1), and then follow the same procedure. Through this iterative process, we can obtain a converged value (~28 meV) of trion binding energy that is in good agreement with the previously reported theoretical value (~26 meV)^32^. Equation (1) and the trion binding energy of 28 meV are then used to plot the electron density as a function of the plasma treatment time (Fig. 3c). The result indicates that sufficient charge neutralization (∆*n*_el_/*n*_el_ ≈ 85.3%) has been achieved in 1L-MoS_2_ via chlorine plasma doping treatment.

We find that the plasma power also affects the PL intensity of 1L-MoS_2_ in a similar manner to that of the plasma treatment time. The PL intensity increases linearly with the plasma power because the intensity of plasma emission is proportional to the plasma power (Supplementary Fig. 5). At above a critical plasma power of 5 W, the PL intensity drops precipitously due to the start of etching (Supplementary Fig. 6), as in the case of the plasma treatment time.

### Reversible PL control via post hydrogen plasma treatment

**Figure 4:**
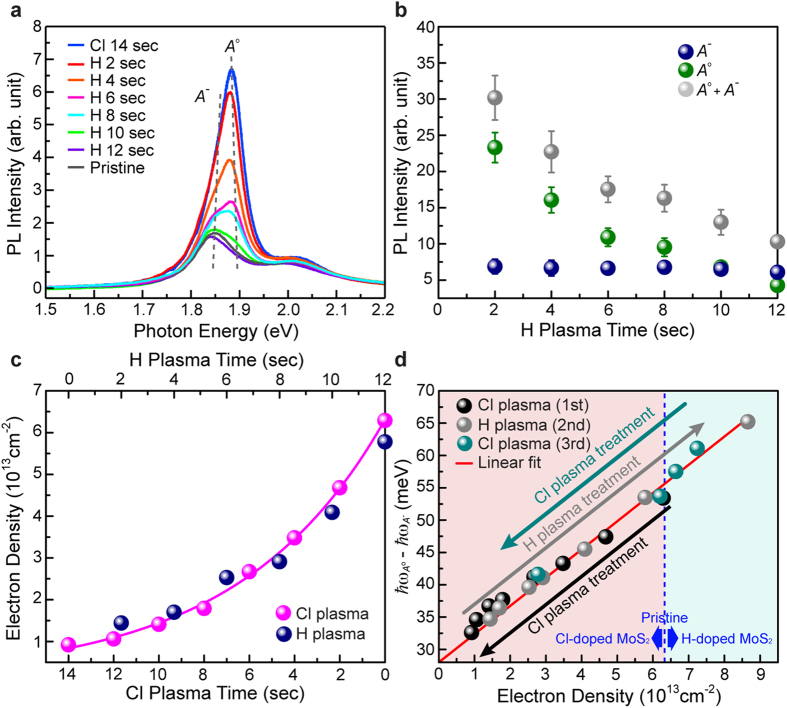
The variation of the PL properties of chlorine-doped 1L-MoS_2_ as increasing the hydrogen plasma treatment time. (**a**) Evolution of the PL spectrum of chlorine-doped 1L-MoS_2_ as the hydrogen plasma treatment time increases. The resonant energies of the excitons (*A*^o^) and the trions (*A*^−^) are indicated with gray dotted lines. (**b**) The PL intensities of the excitons, trions, and their total contribution as a function of the hydrogen plasma treatment time. (**c**) The electron densities plotted as a function of the chlorine plasma treatment time (magenta) and post hydrogen plasma treatment time (blue). The time directions should be noted to be opposite to each other. (**d**) The difference between exciton and trion energies plotted as a function of the electron density for the sequential chlorine plasma (black), hydrogen plasma (gray), and chlorine plasma (cyon) treatments. They follow the same linear relation (red line), indicating that a reversible and repeatable PL control of 1L-MoS_2_ has been successfully achieved in a bidirectional mode.

The most important issue of this study is to suggest a method to reversibly tune the PL of 1L-MoS_2_ using dopants, which has never been reported to date. We have discovered that the enhanced PL intensity of chlorine-doped 1L-MoS_2_ is gradually degraded back to the original emission level as a post hydrogen plasma treatment proceeds (Fig. 4a). By decomposing the PL spectra of 1L-MoS_2_ into those of excitons and trions, we find that the exciton and trion PL intensities follow almost the same time trajectory as in chlorine plasma doping, but with the reverse direction (Fig. 4b). The electron density of chlorine-doped 1L-MoS_2_ is plotted as a function of the hydrogen plasma treatment time (Fig. 4c), and we recognize that the graph fairly resembles that observed for the chlorine plasma doping (Fig. 3c) with a small time scaling (14 sec/12 sec) and reverse time. The difference between exciton and trion resonance energies is also plotted as a function of the electron density, and we find that they are all located close to the theoretical line obtained in the analysis of the chlorine plasma doping (Fig. 4d). All these results consistently indicate that reverse PL control has been successfully achieved in 1L-MoS_2_.

To understand the mechanism of this phenomenon in further details, we carry out XPS measurements of chlorine-doped 1L-MoS_2_ before and after hydrogen plasma treatment (Supplementary Fig. 7). The results show that chlorine species have been completely removed from the sample after hydrogen plasma is applied for a sufficiently long time (~12 sec). This indicates that PL degradation occurs through hydrogen-plasma-assisted chlorine dedoping rather than through an additional (n-type) doping of hydrogen for the PL modulation down to the emission level of as-prepared 1L-MoS_2_, suggesting a possible cycled (i.e., multi-reversible) PL tuning of 1L-MoS_2_. We find that, the binding energies of Mo 3d and S 2p peaks in XPS spectrum blueshifts as chlorine plasma doping proceeds and they can be recovered to the original level of pristine MoS_2_ after post hydrogen-plasma treatment showing good agreement with the previous work^33^ (Supplementary Fig. 8).

The continued hydrogen plasma treatment (at least, up to 20 sec) after the exhaustive chlorine dedoping induces the further PL degradation of 1L-MoS_2_ below the emission level of as-prepared 1L-MoS_2_, while conserving well the structural integrity as seen in the negligible Raman signal variation (Supplementary Fig. 9). It is inferred that this PL degradation results from n-type doping of hydrogen instead of plasma etching, and we are indeed able to restore the PL back to the original level through subsequent chlorine plasma treatment (Fig. 4d and Supplementary Fig. 10), similar to chlorine dedoping reaction via post hydrogen plasma. However, in the case of hydrogen plasma treatment for 30 sec, the Raman signal is destroyed considerably due to hydrogen plasma etching (Supplementary Fig. 9).

### Density functional theory calculations of chlorine doping and dedoping reactions

To gain a deeper understanding of the chemical reactions that are associated with the method described above, we carry out density functional theory (DFT) calculations on the charge transfer between the chlorine (or hydrogen) adatom and 1L-MoS_2_ as well as on the energetics of hydrogen (or chlorine)-plasma-assisted chlorine (or hydrogen) dedoping reaction. The results indicate that a positive charge of 0.323e is transferred from the chlorine adatom (on the top of the sulfur atoms) to 1L-MoS_2_ in the chlorine plasma doping, while a positive charge of 0.151e is transferred from the F_4_TCNQ molecule to 1L-MoS_2_, indicating a superior doping effect of chlorine plasma. The advantage of chlorine plasma doping is manifested even more if we consider the large difference between the molar volumes of a F_4_TCNQ molecule (204.88 Å^3^) and a chlorine atom (20.41 Å^3^). That is, the chlorine plasma doping requires volumetric and gravimetric dopant quantities that are ~21.5 and ~16.7 times as small as those required for chemical doping of F_4_TCNQ, respectively. As a result, only ~14.5% of the adsorption sites in the MoS_2_ surface is occupied by chlorine atoms in the case with the largest PL enhancement (charge neutralization of 86.7%) in this study. From the hydrogen atom adsorbed on top of the sulfur atoms, a negative charge of −0.0366e is transferred to the 1L-MoS_2_. The adsorption energies of the chlorine and hydrogen atoms are estimated to be 35.28 and 37.07 kcal/mol, respectively.

**Figure 5:**
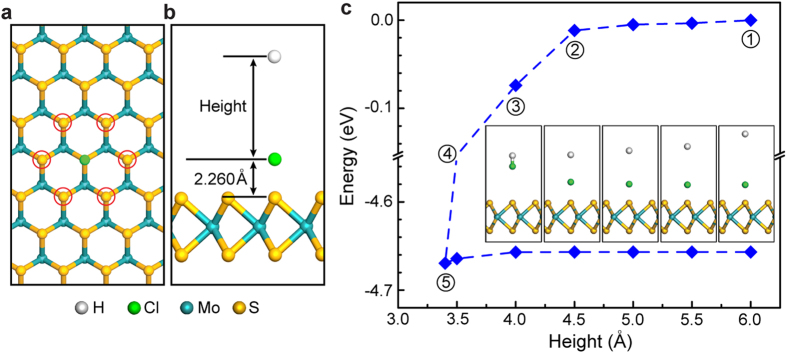
The energetics of hydrogen-plasma-assisted chlorine dedoping reaction in 1L-MoS_2_. (**a**) The optimized structure of chlorine-doped 1L-MoS_2_, with red circles indicating the nearest-neighboring S atoms of the chlorine adatom. (**b**) The reaction path of hydrogen-plasma-assisted chlorine dedoping in chlorine-doped 1L-MoS_2_ where the height is used as a reaction path variable that is defined as the distance of the hydrogen atom from the chlorine adatom in the optimized structure of the chlorine-doped 1L-MoS_2_. (**c**) The variation in the system energy (counterclockwise) for the gradual approach of the hydrogen atom to the chlorine adatom and the subsequent detachment of the hydrogen-chlorine moiety from the MoS_2_ surface. The structures of the sequential stages (1–5) are respectively shown in the small right-to-left figures inside.

Finally, we investigate the energetics of hydrogen-plasma-assisted chlorine dedoping reaction using DFT-based reaction path analysis. For this calculation, the positions of the six nearest-neighboring sulfur atoms of the chlorine adatom (red circles in Fig. 5a) remain fixed to describe the immobilized MoS_2_ substrate, and the height is used as a reaction path variable that is defined as the distance of the hydrogen atom from the chlorine adatom in the relaxed structure of the chlorine-doped 1L-MoS_2_ (Fig. 5b).

The system energy decreases slightly upon the height reduction from 6.0 Å to 4.5 Å. However, as the height decreases further (i.e., as the hydrogen atom approaches the chlorine adatom), the energy of the system starts to drop precipitously, reaching a minimum energy of −4.67 eV at a height of 3.4 Å with the formation of a stable hydrogen-chlorine moiety (Fig. 5c). As the height increases back to the initial value of 6.0 Å from this critical height, the hydrogen-chlorine moiety readily departs from the MoS_2_ surface with negligible energy consumption. Overall, the reaction occurs with almost no energy barrier, and the associated reaction energy is estimated to be −107.4 kcal/mol, which is about three times as large as the adsorption energy of chlorine, suggesting the facile hydrogen-plasma-assisted chlorine dedoping as observed in the experiments. Meanwhile, the chlorine-plasma-assisted hydrogen dedoping reaction takes place with a small energy barrier of 2.88 kcal/mol and a large negative reaction energy of −88.47 kcal/mol (Supplementary Fig. 11), indicating the possible reversible PL tuning of 1L-MoS_2_ in the bidirectional regime, which is consistent with our experimental observations.

The intrinsic sulfur vacancies have been widely detected in the mineral MoS_2_^34^, and hence, we have also investigated the doping case in which the chlorine atoms are adsorbed in the sulfur vacancies. The charge analysis indicates that a positive charge of 0.568e is transferred from this chlorine atom to 1L-MoS_2_, which is remarkably larger than the charge transfer (0.323e) from the chlorine atom on top of the sulfur atom. As a consequence, less surface coverage of chlorine (~8.2%) is required compared to the previous doping case (~14.5%) in order to achieve the charge neutralization of ~85.3%. Thus, it is supposed that the actual surface coverage would lie between 8.2 and 14.5% since the mixed adsorption state exists in 1L-MoS_2_ generally. The adsorption energy of these chlorine atoms is estimated to be −89.3 kcal/mol, which is ~2.5 times as large as in the previous chlorine doping case. In this chlorine doping case, the DFT calculation indicates that the hydrogen-plasma-assisted chlorine dedoping reaction occurs with an energy barrier of 10.08 kcal/mol, and its reaction energy is −46.76 kcal/mol, which is half of the value in the previous chlorine doping case (Supplementary Fig. 12).

## Discussion

Plasma functionalization has been widely used to alter the electronic structures of graphene for band-gap engineering^35^,^36^ and carrier doping^37^,^38^. Here we show that the PL intensity of 1L-MoS_2_ can be drastically enhanced by chlorine plasma functionalization^39^,^40^ via trion-to-exciton transition, and the PL intensity is easily tuned by controlling the plasma treatment time or the plasma power. Theoretical calculations indicate that sufficient charge neutralization (~83%) can be achieved in 1L-MoS_2_ via small chlorine plasma doping, where only 8–15% of the MoS_2_ surface needs to be occupied by chlorine atoms due to their strong electron affinities. This allows a large portion of MoS_2_ surface to remain bare, offering a space for external molecule adsorption and other forms of chemical functionalization, which is necessary to molecular sensing applications. Such conditions are difficult to achieve with conventional chemical doping because full surface coverage of chemical dopants is generally required to obtain sufficient charge neutralization in 1L-MoS_2_. The failure of the complete charge neutralization in this study is caused by the occurrence of MoS_2_ etching, and this limitation can be overcome by optimizing doping condition or using devices that are specially designed for plasma deposition rather than for plasma etching, e.g., equipment containing multi-grids in front of a plasma gun to filter the high energy particles.

It has been difficult to reversibly tune the PL of 1L-MoS_2_ using conventional chemical doping method due to the absence of an appropriate technique to detach the chemical dopants in a controllable way. MoS_2_. Concerning to this issue, we suggest that reversible PL tuning can be easily realized in 1L-MoS_2_ by the application of hydrogen plasma to chlorine-doped 1L-MoS_2_ because the hydrogen plasma efficiently dedopes chlorine adatoms. It is demonstrated that the same mechanism can be applied to chlorine-assisted hydrogen dedoping in 1L-MoS_2_, extending the range of charge doping (i.e., PL control) to a bidirectional regime.

We show that the trion binding energy of 1L-MoS_2_ can be obtained theoretically through an iterative process between equations (1) and (2). The converged value of the trion binding energy is estimated to be of ~28 meV, which is in excellent agreement with a value of ~26 meV obtained via DFT calculation. This theoretical analysis method can be widely used in studies of exciton emission of other 2D materials.

We expect that this cyclically-tunable carrier doping method developed here will serve an important role in fabricating TMD-based light-emitting diodes and photovoltaics (e.g., facile manufacturing of WSe_2_ diodes composed of closely-located hydrogen-doped and chlorine-doped domains)^41^,^42^,^43^ as well as in reducing the contact resistance between the TMD channel and the metal electrodes in FET devices^33^, both of which are emerging issues presenting important challenges. In the current study, we have tested our carrier doping method for chlorine dopant and MoS_2_ only, but this method can be readily extended not only to other halogen dopants and TMDs but also to other two-dimensional materials like graphene.

## Methods

### MoS_2_ Sample Preparation

Mono- and bi-layer MoS_2_ are exfoliated from commercial bulk crystals (SPI supplies Inc.) using a micro-mechanical method and are then transferred onto a silicon substrate capped with a 300 nm thick SiO_2_ layer. The typical size of 1L-MoS_2_ ranges from 2 to 5 μm. As with graphene, the number of MoS_2_ layers is first estimated according to the optical contrast in optical microscopy image and is then confirmed with AFM in a non-contact mode.

### PL and Raman spectroscopies

Micro-PL and Raman spectroscopies of the MoS_2_ samples are performed in the backscattering geometry under ambient condition. The excited radiation is induced by a ND:YAG laser with a central wavelength of 532 nm, and the laser power is kept below 0.45 mW during the measurement to avoid heating and thus possible damaging the samples. The PL emission is collected with a 100X objective and 1800 grooves per millimeter with a spectral resolution of 1.5 cm^−1^, while the Raman emission is collected with the same objective and 2400 grooves per millimeter with a spectral resolution of 0.6 cm^−1^. The PL and Raman spectra are measured using an air-cooled charge-coupled device.

### Chlorine and Hydrogen Plasma treatments

Chlorine and hydrogen plasma treatments are performed on the MoS_2_ samples using an electron-cyclotron resonance-reactive ion etcher (Plasmalab 100 ICP System, Oxford Inst.) We carefully optimize the DC bias to control the RF power by keeping the inductive coupled plasma (ICP) power, the flow rate, and the pressure fixed. The ICP power is fixed to 100 W, and the flow rate of chlorine and hydrogen gases and the pressure are maintained at 80 sccm and 20 mTorr, respectively. The chamber in our experiments is always kept to 25 ^o^C, and before each run, we clean the chamber by using oxygen plasma for 5 min, and the chamber is subsequently filled with chlorine or hydrogen gas for another 5 min at the desired pressure without samples.

### First-principles calculations

All of the DFT calculations are performed within generalized gradient approximations (GGA) based on Perdew-Burke-Ernzerholf exchange-correlation functional as implemented in the ATK software package^44^. The mesh cutoff for electrostatic potentials is set to 200 Rydberg and double-ζ polarized basis sets are used as the local atomic numerical orbitals. The systems are relaxed until the residual forces and stresses are below 0.03 eV Å^−1^ and 0.03 eV Å^−2^, respectively. Then, the energies and the Bader charges are evaluated using this optimized structure. A 6 × 6 × 1 supercell is used for 1L-MoS_2_. A 3 × 3 × 1 Monkhorst Pack grid is used for k-space sampling in the geometric optimization while a 4 × 4 × 1 grid is used for k-space sampling in the Bader charge analysis^45^ and the internal energy calculation.

### Analysis of exciton and trion emission intensities

The excition PL and trion PL in 1L-MoS_2_ are analyzed within the framework of a three-energy-level model. In this model, the populations of the excitons and the trions are expressed as^19^


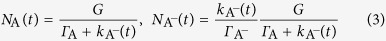


where 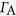 and 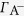 are the decay rates of the excitons and trions, respectively; 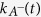
is the formation rate of trions from excitons at plasma treatment time *t*; and *G* is the optical generation rate of excitons. In this analysis, 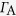, 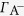, and 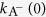 are set to 0.002, 0.02, and 0.5 ps^−1^ on the basis of previously reported values that were obtained from transient absorption measurments^46^.

The PL intensities of the excitons (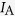) and trions (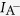) are proportional to the populations of the excitons and trions, respectively, and they can be expressed as follows:


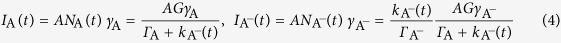


Where *A* is a proportionality constant expressing the collection efficiency of the luminescence, and 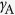 and 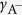 are the radiative decay rates of excitons and trions, respectively. In this study, 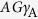 and 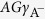 are determined to be 20 and 1.52, respectively, as the best-fit parameters for the experimental results, as can be seen in Fig. 3b.

### The relationship among the trion spectral weight, carrier density, and trion binding energy

On the basis of the mass action law, the equation relating the concentrations of trions (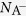) and excitons (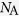) and the charge carrier densiy (*n*_el_) in TMDs can be drawn as follows:


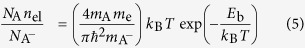


where 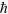
is the reduced Planck’s constant; *k*_B_ is the Boltzmann constant; *T* is the temperature; *E*_b_ is the trion binding energy; and *m*_e_, 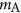, and 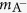 are the effective electron, excition, and trion eneries, respectively. In the application for 1L-MoS_2_, *m*_e_, 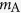, and 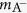 are taken as 0.35*m*_0_, 0.8*m*_0_, and 1.15*m*_0_, repsecitvely, where *m*_0_ is the mass of free electrons^10^.

Using equations (4) and (5), the relationship between the trion spectral weight and the carrier density in 1L-MoS_2_ can be obtained as follows:


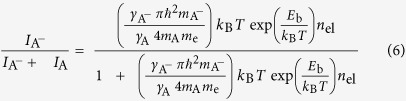


By measuring the trion PL spectral weight and taking 20 meV as the initial value that was guessed for the trion binding energy, we estimate the electron density using equation (6) for each plasma treatment time. Then, we plot the difference between the exciton and triton energies as a function of the electron density using equation (2). Finally, we obtain the intercept of the fitted line for their correlation, which determines the trion binding energy. We compare this trion binding energy with the value that was initially guessed, and if their difference is larger than 1 meV, we input the value that was obtained as the new initial value for the trion binding energy, and perform the same process again. Through this iterative process, we obtain the converged trion binding energy for 1L-MoS_2_.

## Additional Information

**How to cite this article**: Kim, Y. *et al.* Plasma functionalization for cyclic transition between neutral and charged excitons in monolayer MoS_2_. *Sci. Rep.* **6**, 21405; doi: 10.1038/srep21405 (2016).

## Supplementary Material

Supplementary Information
