# Supplementary material for: Plasma functionalization for cyclic transition between neutral and charged excitons in monolayer MoS2
Source: Sci Rep. 2016 Feb 22;6:21405. doi: 10.1038/srep21405 (PMC4761959; doi:10.1038/srep21405)
Supplement: Supplementary Information [file srep21405-s1.doc]

Supporting Information for

**Plasma functionalization for cyclic transition between neutral and charged excitons in monolayer MoS_2_**

Y. Kim^+^, Y. I. Jhon^+^, J. Park, C. Kim, S. Lee, and Y. M. Jhon^*^

Sensor System Research Center, Korea Institute of Science and Technology, Seoul 136-791, Republic of Korea

^+^Y. Kim and Y. I. Jhon are equally contributed in this work.

1. **AFM analysis of Cl-doped and pristine 1L-MoS_2_**


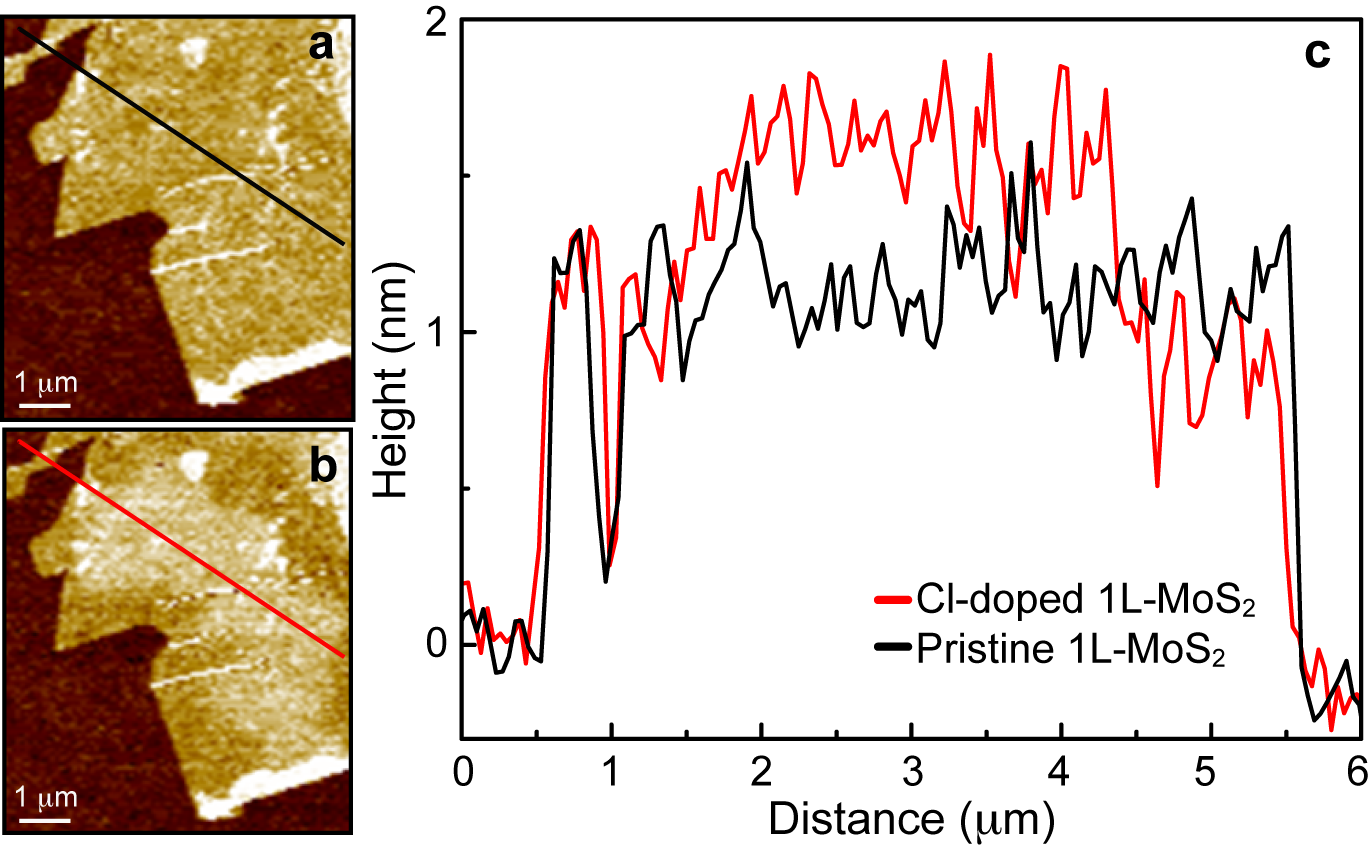


**Figure S1. (a, b)** AFM images of pristine and Cl-doped 1L-MoS_2_ samples and **(c)** corresponding height profiles measured along the black and red solid lines in the AFM images.

1. **Environmental stability of Cl-doped 1L-MoS_2_**


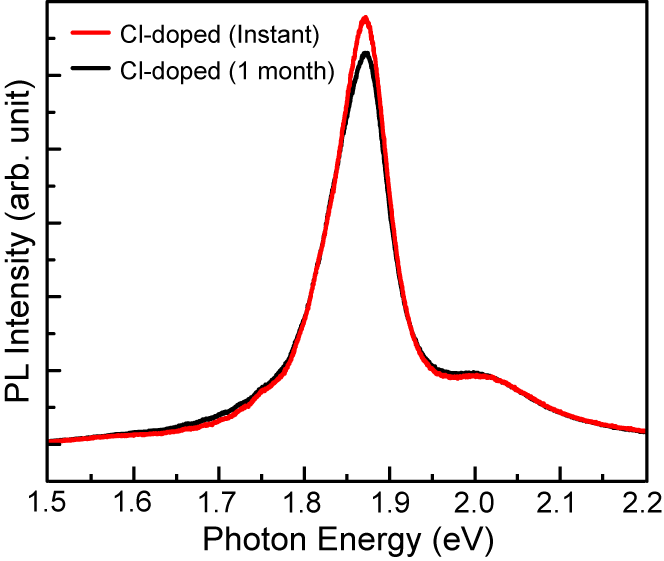


**Figure S2.** PL spectral change of Cl-doped 1L-MoS_2_ after one month.

1. **The evolution of Raman spectra with Cl plasma time**


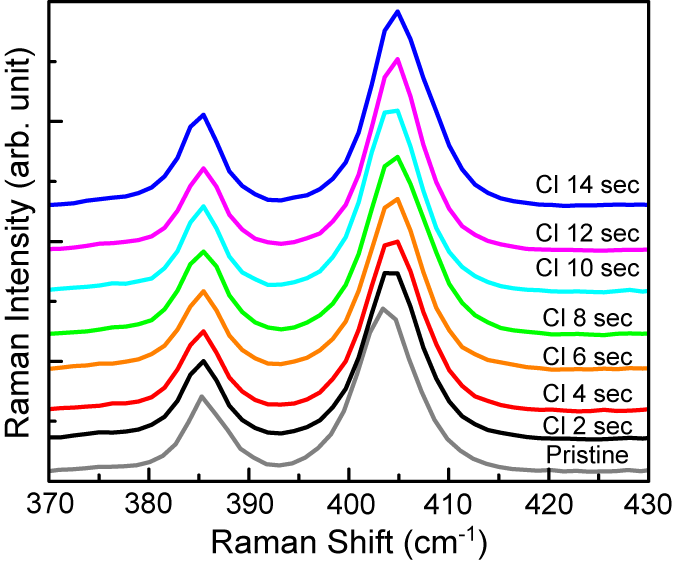


**Figure S3.** The evolution of $\text{E}_{\text{2g}}^{1}$(in-plane) and $\text{A}_{\text{1g}}$(out-of-plane) Raman modes as Cl plasma treatment time increases.

1. **PL and Raman spectra of 1L-MoS_2_ with excessive Cl plasma time**

**
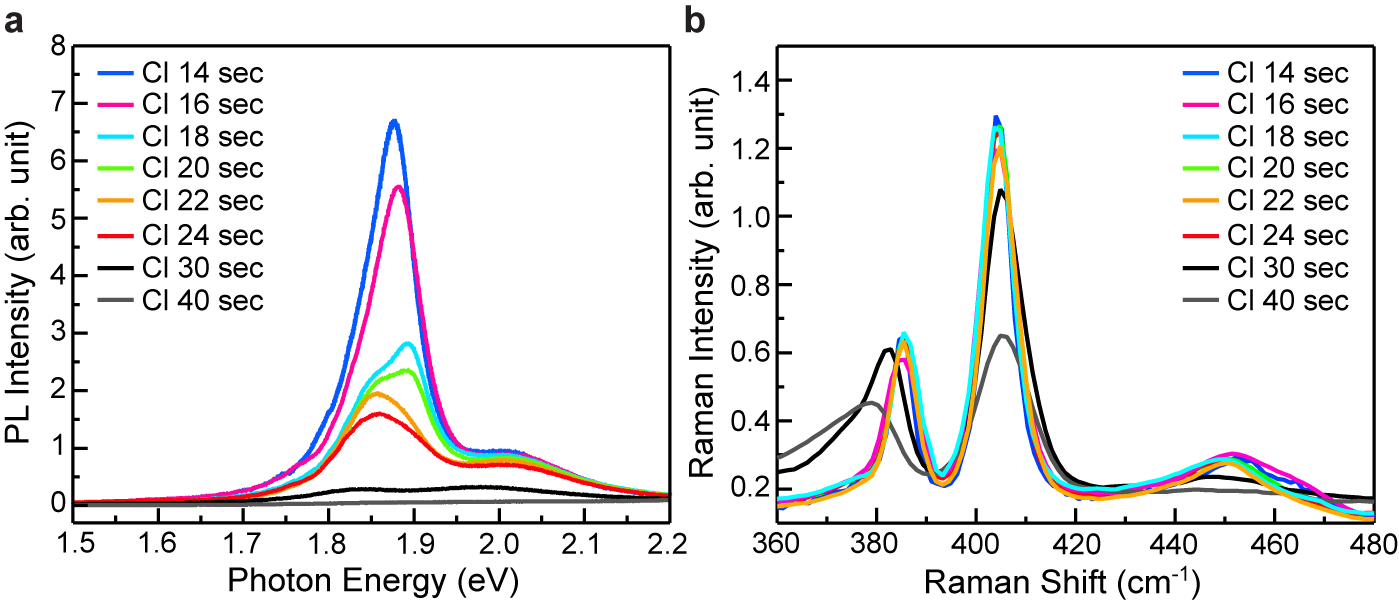
**

**Figure S4.** (**a**) PL and (**b**) Raman spectra of 1L-MoS_2_ obtained after a threshold Cl plasma treatment time of 14 sec.

1. **Plasma power effects on the PL and Raman spectra of 1L-MoS_2_ during Cl plasma treatment**


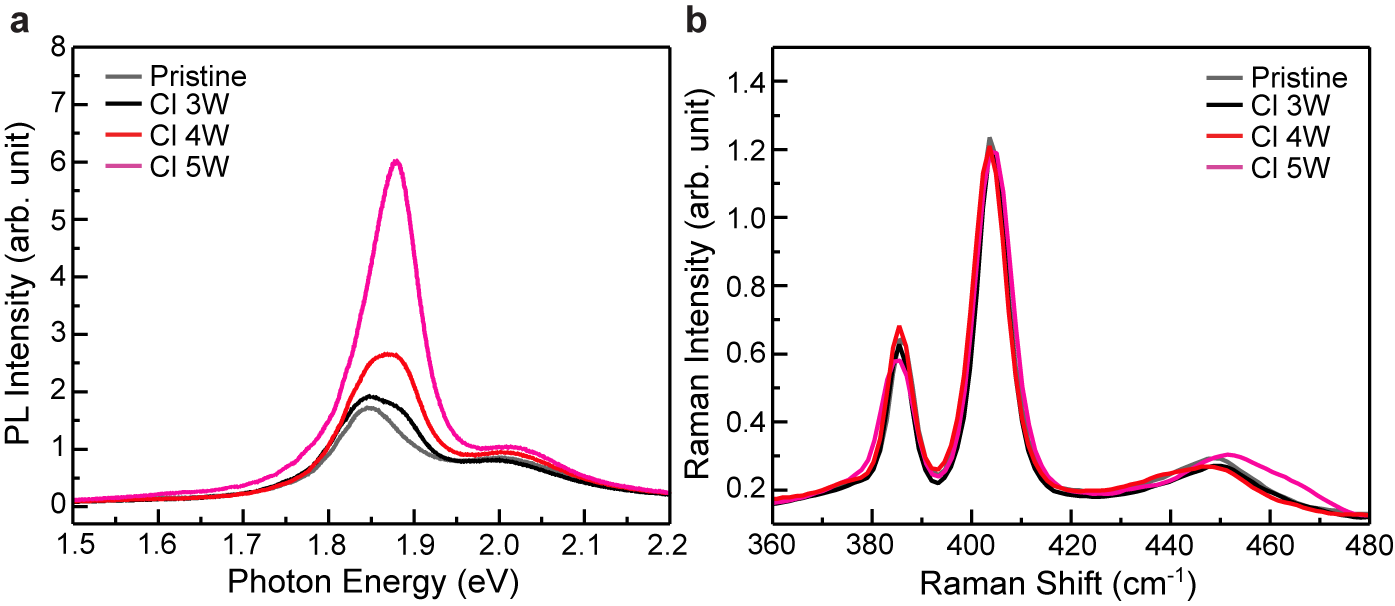


**Figure S5** (**a**) PL and (**b**) Raman spectra of 1L-MoS_2_ with increasing the Cl plasma power.

1. **PL and Raman spectra of 1L-MoS_2_ with excessive Cl plasma power**


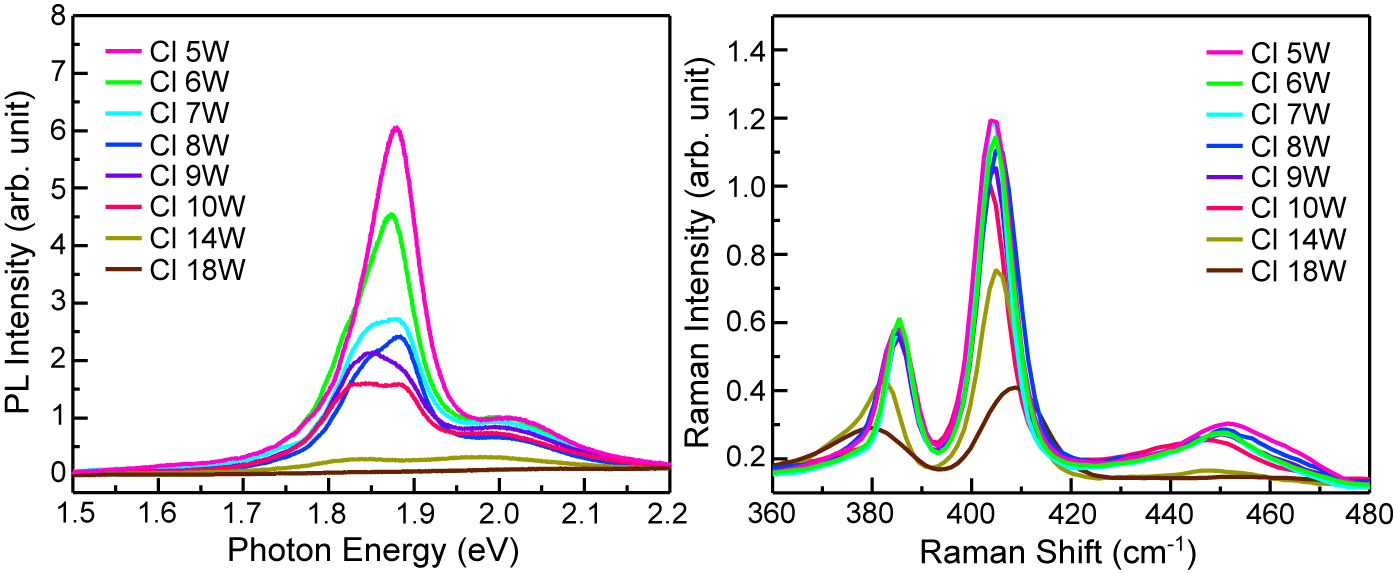


**Figure S6.** (**a**) PL and (**b**) Raman spectra of 1L-MoS_2_ obtained after a threshold Cl plasma power of 5 W.

**7. XPS analysis of Cl doped 1L-MoS_2_ before and after post H plasma treatment**


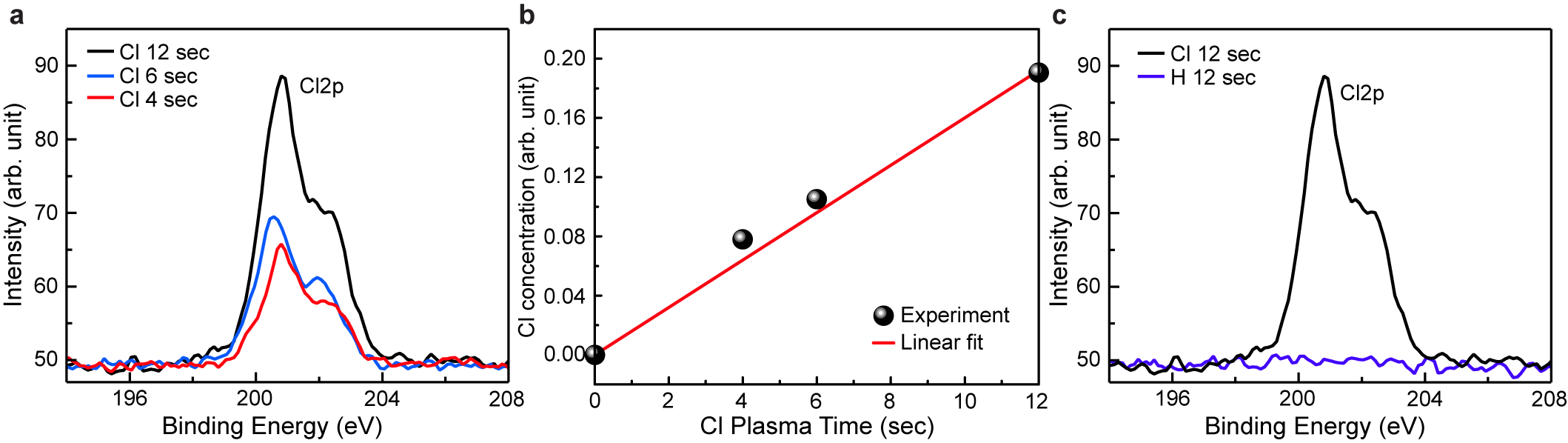


**Figure S7.** (**a**) Cl-2p XPS spectra measured for 1L-MoS_2_ after different Cl plasma treatment time. (**b**) The variation of Cl concentration plotted as a function of the Cl plasma treatment time. (**c**) Cl-2p XPS spectra of Cl-doped 1L-MoS_2_ measured before and after a sufficient amount of post H plasma treatment.

**8. The binding energies of Mo 3d and S 2p peaks in pristine, Cl-doped, and H-doped 1L-MoS_2_**

**
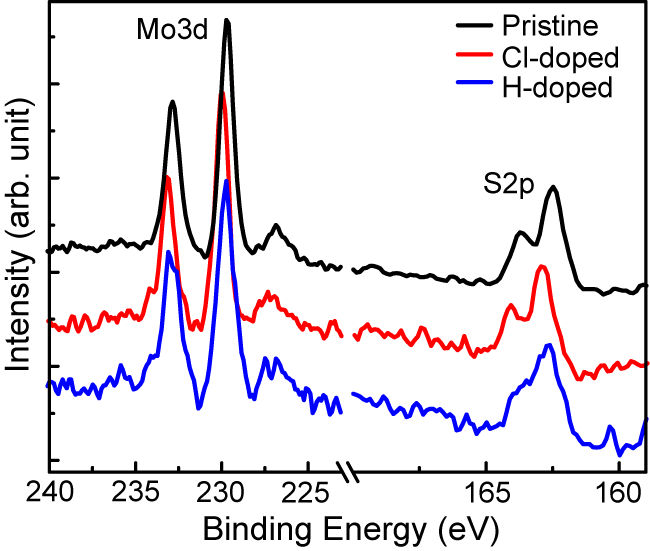
**

**Figure S8.** Mo 3d and S 2p binding energies from XPS measurements of pristine, Cl-doped, and H-doped 1L-MoS_2_.

**9. PL and Raman spectra of Cl-doped 1L-MoS_2_ measured after long H plasma treatment**


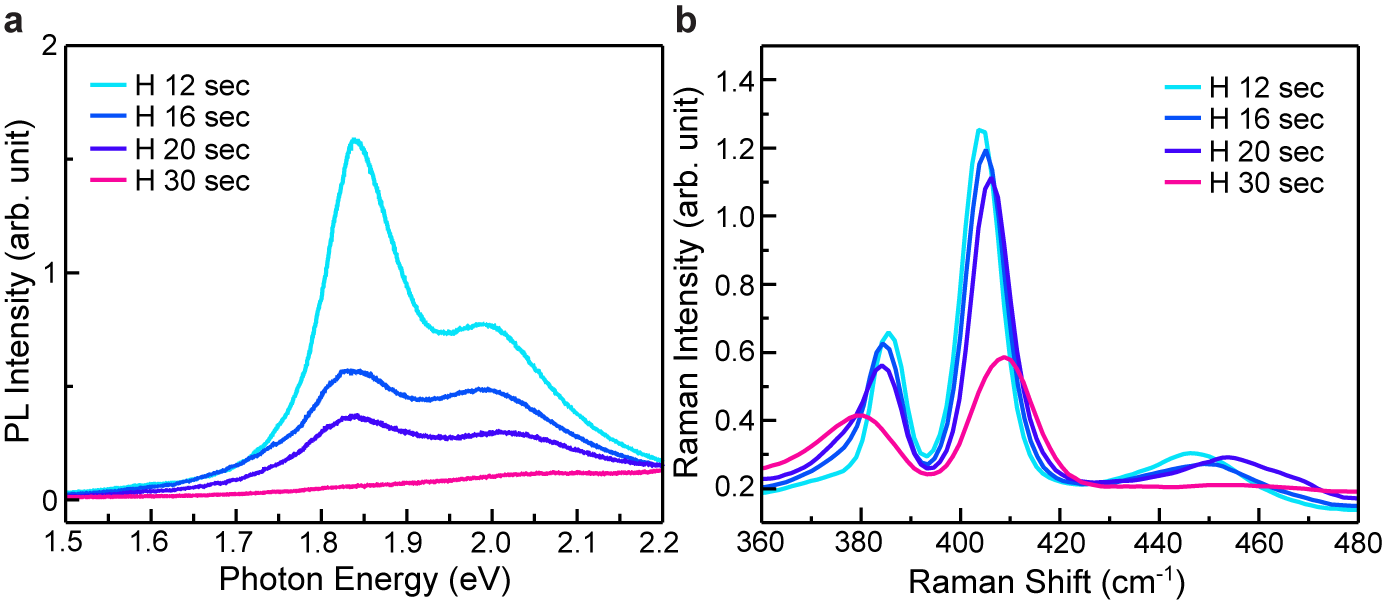


**Figure S9.** (**a**) PL and (**b**) Raman spectra of Cl-doped 1L-MoS_2_ obtained after the H plasma treatment time above 12 sec.

**10. PL spectra of H-doped 1L-MoS_2_ with increasing the Cl plasma treatment time**


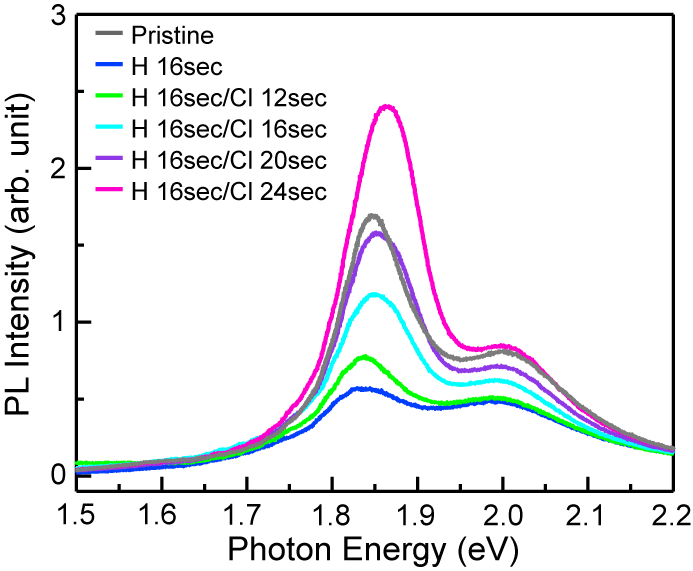


**Figure S10.** Evolution of the PL spectrum of H-doped 1L-MoS_2_ as the subsequent Cl plasma treatment proceeds.

**11. The energetics of Cl-plasma-assisted H-dedoping reaction: the H adatoms adsorbed on top of the S atoms of 1L-MoS_2_**


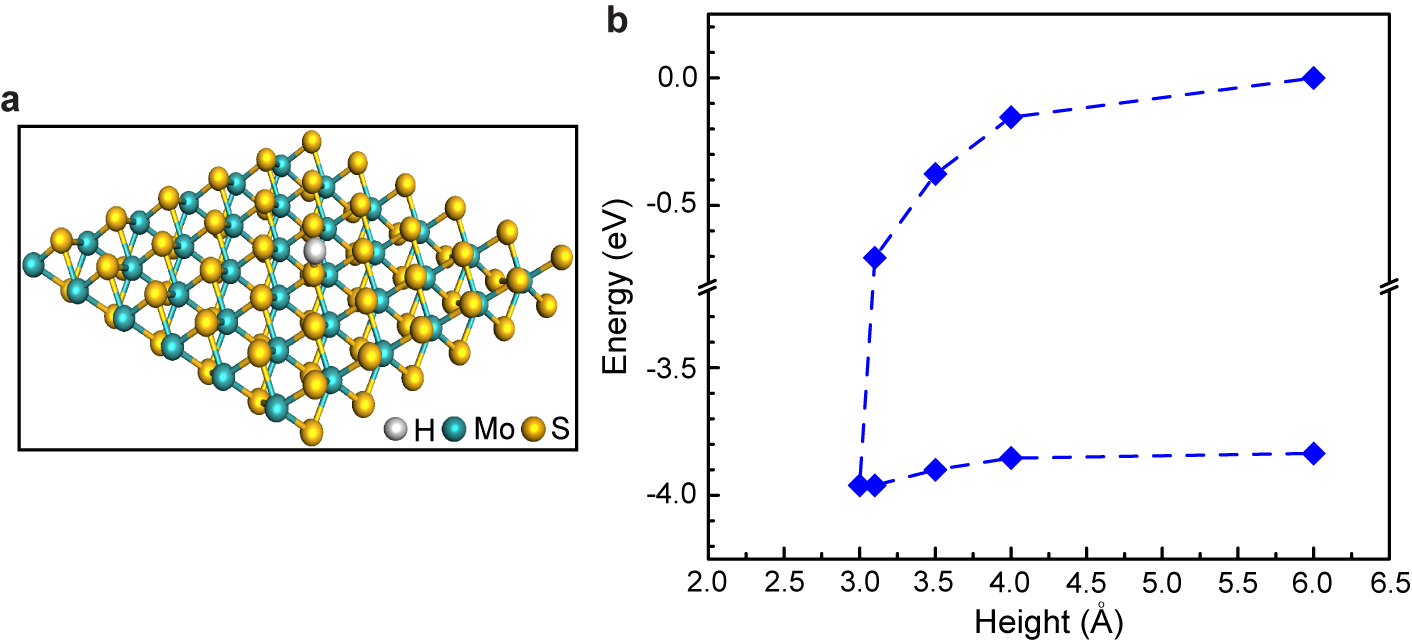


**Figure S11. The energetics of H-plasma-assisted Cl dedoping reaction in 1L-MoS_2_ (the H adatoms on top of the S atoms).** (**a**) The optimized structure of H-doped 1L-MoS_2_ in which the H atoms are adsorbed on top of the S atoms. (**b**) The variation in the system energy (counterclockwise) for the gradual approach of the Cl atom to the H adatom and the subsequent detachment of the H-Cl moiety from the MoS_2_ surface. Here, the height is used as a reaction path variable which is defined as the distance of the Cl atom from the H adatom in the optimized structure of H-doped 1L-MoS_2_.

**12. The energetics of H-plasma-assisted Cl-dedoping reaction: the Cl adatoms adsorbed in the S vacancies of 1L-MoS_2_**

**
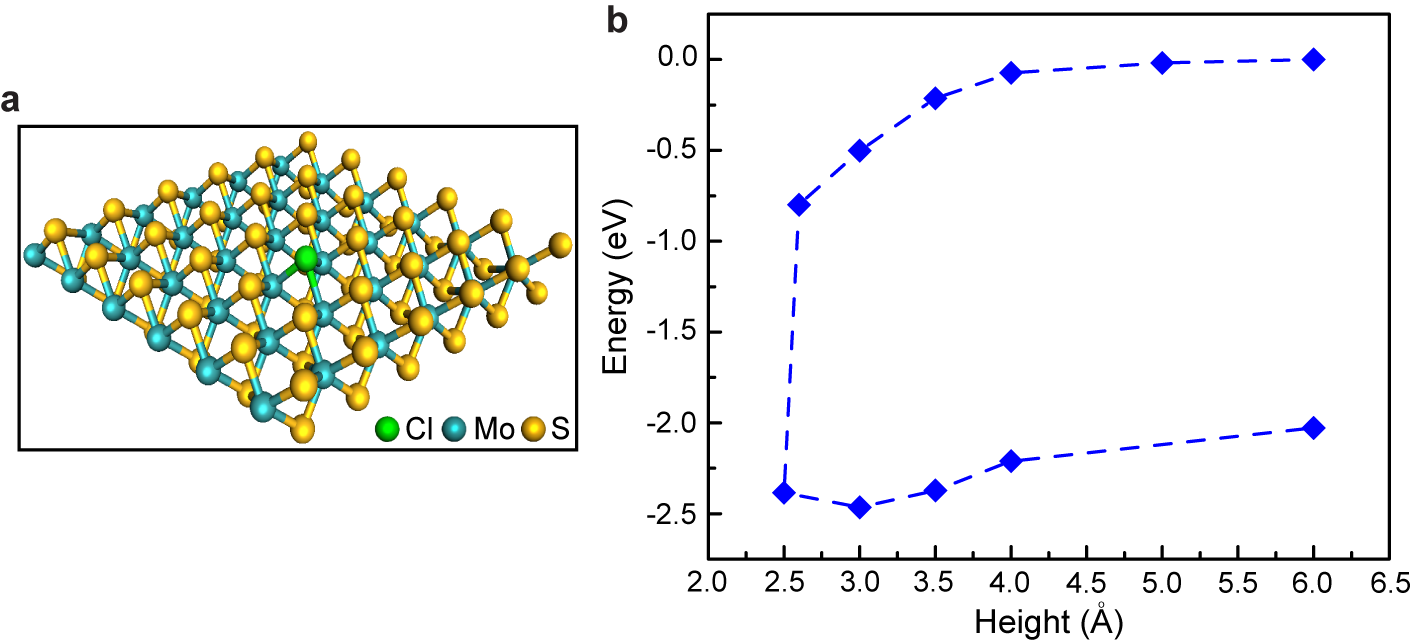
**

**Figure S12. The energetics of H-plasma-assisted Cl dedoping reaction in 1L-MoS_2_ (the Cl adatoms in the S vacancies).** (**a**) The optimized structure of Cl-doped 1L-MoS_2_ in which the Cl atoms are adsorbed in the S vacancies of 1L-MoS_2_. (**b**) The variation in the system energy (counterclockwise) for the gradual approach of the H atom to the Cl adatom and the subsequent detachment of the H-Cl moiety from the MoS_2_ surface. Here, the height is used as a reaction path variable which is defined as the distance of the H atom from the Cl adatom in the optimized structure of Cl-doped 1L-MoS_2_.
